# Diet and Leukocyte Telomere Length in a Population with Extended Longevity: The Costa Rican Longevity and Healthy Aging Study (CRELES)

**DOI:** 10.3390/nu13082585

**Published:** 2021-07-28

**Authors:** Edward A. Ruiz-Narváez, Ana Baylin, Jorge Azofeifa, Alejandro Leal, Luis Rosero-Bixby

**Affiliations:** 1 Department of Nutritional Sciences, School of Public Health, University of Michigan, Ann Arbor, MI 48109, USA; abaylin@umich.edu; 2 Department of Epidemiology, School of Public Health, University of Michigan, Ann Arbor, MI 48109, USA; 3 Escuela de Biología, Universidad de Costa Rica, Apartado, San Pedro, San José 11501, Costa Rica; azofeifa.navas@gmail.com (J.A.); alejandro.leal@ucr.ac.cr (A.L.); 4 Centro Centroamericano de Población, Universidad de Costa Rica, Apartado, San Pedro, San José 11501, Costa Rica; lrosero@mac.com

**Keywords:** longevity, aging, diet, telomeres, Costa Rica, Nicoya

## Abstract

Elderly Costa Ricans have lower mortality rates compared to their counterparts from developed countries. Reasons for this survival advantage are not completely known. In the present study, we aimed to identify dietary factors associated with leukocyte telomere length (LTL), a marker of biologic aging, in the elderly population of Costa Rica. We conducted prospective analysis in 909 participants aged 60+ years from the Costa Rican Longevity and Healthy Aging Study (CRELES). We used a food frequency questionnaire to assess usual diet. We calculated dietary patterns using Principal Component Analysis (PCA). We used generalized linear models to examine the association of dietary patterns and food groups with leukocyte telomere length. We found two major dietary patterns explaining 9.15% and 7.18% of the total variation of food intake, respectively. The first dietary pattern, which represents a traditional Costa Rican rice and beans pattern, was more frequent in rural parts of the country and was positively associated with baseline LTL: β (95% CI) = 42.0 base-pairs (bp) (9.9 bp, 74.1 bp) per one-unit increase of the traditional dietary pattern. In analysis of individual food groups, intake of grains was positively associated with baseline LTL: β (95% CI) = 43.6 bp (13.9 bp, 73.3 bp) per one-serving/day increase of consumption of grains. Our results suggest that dietary factors, in particular a traditional food pattern, are associated with telomere length and may contribute to the extended longevity of elderly Costa Ricans.

## 1. Introduction

Elderly Costa Ricans, particularly males, have one of the lowest mortality rates in the world [1], outperforming even high-income countries including the U.S. [2]. This survival advantage is more pronounced among elderly Costa Rican males living in the northwest region of Nicoya, which has been recognized as one of five locations in the world (Sardinia in Italy, Okinawa in Japan, Loma Linda in California U.S., Nicoya in Costa Rica, and Ikaria in Greece) with exceptional longevity [3]. Life expectancy of a 60-year old Nicoya male (24.3 years) is more than two years higher than other Costa Rican men (22.0 years) [4], and almost two years higher over Okinawans (22.6 years) [4]. In addition, a 60-year old Nicoyan male has a probability of 4.8% of becoming a centenarian, four times the probability of other Costa Rican men, more than twice that of Okinawans, and almost seven times that of Sardinia [4]. Supportive socio/familial environments, as well as lifestyle factors correlate with the Costa Rican low mortality. For example, most older Costa Ricans live in households of three or more members, primarily with adult children, compared to only about 25% in the U.S. [2].

Diet may play a role explaining the extended longevity of elderly Costa Ricans. For example, beans, a staple food and a major source of protein in Costa Rican adults, have been found associated with low cardiometabolic risk [5] and decreased incidence of non-fatal myocardial infarction in Costa Rica [6]. The diet of elderly Nicoyans in particular is rich in plant-based foods (e.g., legumes, cereals) followed by animal-origin protein, resulting in a high-fiber, low-glycemic index diet [4,7]. It is noteworthy that white rice, which generally is consumed together with beans in Latin American countries, may confer higher risk of adverse metabolic outcomes [5,8]. As beans and white rice are then part of a traditional food pattern in Costa Rica, dietary pattern analysis offers a powerful tool to examine foods that may contribute to extended longevity in elderly Costa Ricans.

Telomeres—repetitive DNA sequences at the chromosome ends—shorten with age [9,10]. Shorter leukocyte telomere length (LTL) have been found associated with higher all-cause mortality (see recent meta-analysis [11]) and, in particular, with adverse cardiovascular outcomes [12,13,14]. Elderly Costa Ricans living in the Nicoya region seems to have longer LTL compared to the rest of the country [15]. In addition, LTL is a predictor of mortality among elderly Costa Ricans, although its association is largely attenuated after adjustment for age and sex [16]. In the present study, we used data from the Costa Rican Longevity and Healthy Aging Study (CRELES) to identify foods and food patterns associated with LTL. We postulate that the traditional Costa Rican diet pattern may be associated with longer LTL.

## 2. Methods

### 2.1. Study Population

All analyses in the present work are based on publicly available and de-identified data from the Costa Rican Longevity and Healthy Aging Study (CRELES). CRELES is a nationally representative longitudinal survey of health and life course experiences of 2827 Costa Ricans ages 60 and over in 2005 [17]. CRELES is being conducted by the Central America Population Center at the University of Costa Rica. The main study objective was to determine the length and quality of life, and its contributing factors in the elderly of Costa Rica. The sample was drawn from Costa Rican residents in the 2000 population census who were born in 1945 or before, with an over-sample of the oldest-old (ages 95 and over). Costa Rica has a public universal health care system that is administered by the autonomous Costa Rican Social Security Fund known as CCSS (Spanish acronym for “Caja Costarricense de Seguro Social”). The CCSS divides the national territory in 102 first-level health areas. CRELES took a systematic subsample of 60 health areas, covering 59% of the Costa Rican territory.

### 2.2. Data Collection

A baseline household interview was conducted by trained personnel between November 2004 and September 2006. Participants were asked questions on a broad range of topics including self-reported physical health, psychological health, living conditions, dietary information, health behaviors, health care utilization, social support, and socioeconomic status. The data also include measured and observed health indicators as well as mortality information provided by surviving family members. The baseline interview yielded a response rate of 85% from the located survivors. Among those interviewed, 95% provided a blood sample, 92% collected overnight urine, and 91% had all anthropometric measures. A second wave of interviews was conducted from October 2006 to July 2008, with 2364 surviving and contacted participants to update questionnaire data and to collect a second round of blood and urine samples. A third interview was carried out from February 2009 to January 2010, with 1855 surviving and contacted participants. All de-identified CRELES databases from waves 1, 2, and 3 are public and available from The National Archive of Computerized Data on Aging of the University of Michigan: http://www.icpsr.umich.edu/icpsrweb/NACDA/series/386 (accessed on 25 July 2019).

### 2.3. Dietary Information

Data on the diet of participants were collected with an abbreviated version of a food frequency questionnaire (FFQ) that was developed and validated to use in the adult population of Costa Rica [18,19]. The abbreviated FFQ (27 food items) was created using stepwise regression to select the minimum number of food items that maximizes the explained variance of a number of nutrients of interest to CRELES (Supplementary Table S1).

### 2.4. Leukocyte Telomere Length (LTL)

Description of methods to measure LTL can be found in [20]. Briefly, LTL was assayed using quantitative polymerase chain reaction (Q-PCR) in triplicates in 1261 CRELES participants who donated a blood sample during wave 1 or wave 2 of interviews. The average inter-assay coefficient of variability was 0.037 [20]. LTL measurements were carried out in two batches. A validation study was conducted in 29 randomly selected DNA samples, which were assayed in both batches. Correlation coefficient between both measurements from both batches of the 29 DNA samples was 0.94 [20]. Mean LTL is expressed as the relative ratio of telomere to a single-copy gene (i.e., human beta-globin) (T/S ratio). The T/S ratio was transformed to base pairs using the equation: base-pairs (bp) = 3274 + 2413 × (T/S) based on [21]. De-identified LTL data are public and available from https://journals.plos.org/plosone/article?id=10.1371/journal.pone.0223766 (accessed on 11 October 2019).

### 2.5. Statistical Analysis

We used SAS software version 9.4 (SAS Institute, Cary, NC, USA) to carry out statistical analysis. All analyses are based on data from 909 CRELES participants who provided a blood sample and have LTL measures from both the first (2004–2006) and second wave of interviews (2006–2008). Questionnaire data including dietary information was taken from the first wave of interviews. Food items were classified into 21 foods groups (Supplementary Table S2) based on previous studies in the Costa Rican population [22,23]. Consumption of the different food groups was adjusted by total energy intake using the residuals method [24]. We used principal component analysis (PCA) to identify factors explaining variation of energy-adjusted food-group intake. We kept two factors based on early findings of two major dietary patterns in Costa Rican adults [22]. The two factors were then orthogonally rotated to facilitate interpretation. We tested for linear trends of covariates across quintiles of the dietary patterns by using the median value of each dietary pattern quintile as a continuous explanatory variable in regression analysis.

We used generalized linear models to examine cross-sectional and prospective associations of baseline (i.e., wave 1) dietary patterns with LTL from wave 1 and wave 2, respectively. We also examined the association of dietary patterns with LTL change between both waves of interviews. We first ran a basic model adjusting for age in years (continuous), sex, and energy intake in kilocalories (continuous). We then added covariates related to blood collection (season of blood collection), DNA isolation (number of years that DNA was stored at 4 Celsius degrees; whether DNA was extracted from blood cells that were fresh or stored <12 months) and LTL measurement (lot of the LTL assay: 2010 or 2014). These covariates have been found associated with LTL in CRELES [20]. In our final multivariate model, we added covariates related to data collection (interviewer), or potential confounders associated with dietary patterns: place of residence (Nicoya vs. rest of the country, rural vs. urban), education level (none, elementary (1–6 years), high school (7–11 years), more than high school), smoking (never, past, current), alcohol drinking (never, past, current), regular exercise (yes, no), self-rated health (excellent, very good, good, fair, poor), household wealth (high, medium, low), and self-reported diagnosis of diabetes (yes, no). In addition, prospective analyses were done with and without adjustment for baseline LTL.

We further examined association of individual food groups with telomere length using generalized linear models. We chose those food groups with loadings (i.e., correlations) of at least 0.30 in absolute value with any dietary pattern found significantly associated with LTL. The selected foods/food groups were jointly analyzed in a single model adjusting for all covariates from the final multivariate model above.

## 3. Results

Table 1 shows loadings of the 21 food groups with the two PCA dietary patterns (Factor 1 and Factor 2). Factor 1 was characterized (|loadings| ≥ 0.3) by higher intakes of grains and legumes; and lower consumption of dairy products, dressings, fruits, fish, and sweets/desserts. Factor 2 was represented by higher intake condiment, red meat, soybean cooking-oil, spread, and fried food, and lower use of hardened palm oil for cooking. PCA factor 1 and factor 2 explained 9.15% and 7.18% of food group intake variation, respectively. Factor 1 seems to represent a traditional Costa Rica rice and beans diet pattern.

Table 2 shows characteristics of CRELES participants by quintiles of the two dietary patterns. Subjects with higher scores of the factor-1 dietary pattern tended to be younger and males, to have longer telomeres at baseline, to live in Nicoya and rural places, and to be current smokers. They also had less years of education, lower prevalence of self-reported diabetes, and low household wealth. Individuals with higher scores of the factor-2 dietary pattern were more likely to be women, current alcohol drinkers, to practice regular exercise, tended to self-rate their health good or better, and had high household wealth. They were also less likely to live in Nicoya and rural places.

Table 3 shows results of association analyses of the dietary patterns with LTL. We found a positive association between the factor-1 dietary pattern and baseline LTL. Each one-unit increase of this diet pattern was associated with an increase of 32.2 bp (95% CI = 1.5 bp, 62.9 bp) and 42.0 bp (95% CI = 9.9 bp, 74.1 bp) of LTL in the basic and more adjusted multivariate model 2 (MV2), respectively. This same dietary pattern was also positively associated with LTL from the second wave of interviews, although the association disappeared after adjustment for baseline LTL. No association was found between the factor-1 dietary pattern and change of LTL. The factor-2 dietary pattern was not associated with LTL or change of LTL.

Table 4 shows results of the joint association analyses of food groups with LTL. We selected those groups with |loadings| ≥ 0.3 with PCA factor 1 (i.e., the dietary pattern associated with LTL). Intake of grains was positively associated with baseline LTL; β (95% CI) = 43.6 bp (13.9 bp, 73.3 bp) per one-serving/day increase of grains. No association was observed between any food group and LTL from the second wave of interviews (with or without adjustment for baseline LTL) or change of LTL.

## 4. Discussion

In the present study, we conducted dietary pattern analysis to identify food factors that may be associated with longer LTL in elderly Costa Ricans, a population with extended longevity [1,2,4]. We found that a traditional Costa Rican diet pattern—characterized by high intake of grains and legumes—was associated with longer LTL both at baseline (i.e., first wave of interviews) and at the second wave of interviews. However, the association of this dietary pattern with LTL at the second wave of interviews disappeared after adjustment for baseline LTL. There was also no association with LTL change between both waves of interviews. This lack of prospective association with LTL may be due to the short time interval between the two waves of interviews (mean = 1.8 years, range = 1.4 years to 3.2 years), that would not allow for big changes of LTL from wave 1 to wave 2. In fact, both LTL measurements were significantly correlated; r = 0.56 (P < 0.001) (Supplementary Figure S1).

Three recent systematic reviews have examined the epidemiological evidence about the association of dietary patterns with LTL [25,26,27]. Although the populations from these studies are not necessarily comparable to ours—they are mostly of European ancestry and younger that participants in CRELES—they may reveal some findings relevant to our present study. The most consistent association found by these reviews was between higher adherence to the Mediterranean diet and longer telomeres [25,26,27]. The Mediterranean diet is characterized by high consumption of plant-based foods and monounsaturated fat from olive oil, moderate consumption of fish, seafood and fermented dairy products, and low consumption of red and processed meats, and sweets [28]. In addition, the Mediterranean diet has been found to be protective against cardiovascular disease [29,30,31]. Although the Costa Rican rice and beans dietary pattern and the Mediterranean diet represent different regional traditional cuisines, some similarities emerge, such as their reliance on plant-based foods, and low consumption of red meats and sweets. It is noteworthy that despite the consistent association of the Mediterranean diet with longer telomeres, the results of its individual food components are less consistent [27]; suggesting the importance of an overall healthy dietary pattern to telomere maintenance.

Our analysis of individual food groups found that consumption of grains—which includes white rice and is a marker of the Costa Rican traditional diet—was associated with longer baseline LTL. Beans, another traditional Costa Rica food, was not associated with LTL. These results seem to be counterintuitive as consumption of white rice is associated with higher risk of adverse cardiometabolic outcomes [5,8], and intake of beans is associated with lower cardiometabolic risk [6]. We note that the reduced FFQ that was used did not differentiate between white and brown rice, the latter being found to be cardioprotective [8]. In addition, although consumption of beans and rice were moderately correlated in this study (energy-adjusted correlation = 0.49), rice showed a wider range of variation (range = 0 servings/day to 4.6 servings/day) compared to beans (range = 0 servings/day to 3.7 servings/day). In addition, less subjects reported low intake (<1 servings/day) of rice (17%) compared to consumption of beans (41%). This suggests that, at least in the CRELES sample and given the limitations of the reduced FFQ that was used, intake of rice may be a better proxy of the Costa Rican traditional diet. It is possible that the reduced FFQ did not capture enough variation of consumption of beans to identify an association with LTL.

Besides grains, no other food group was associated with LTL in our population. To date, the evidence regarding the relationship of LTL with consumption of grains, or any other food group, is unconclusive. The most recent systematic reviews and meta-analyses of epidemiological studies have shown no consistent associations of any food group with LTL [26,27,32,33]. Heterogeneity of these largely observational studies may explain in part the lack of consistent associations of diet with LTL.

The association of a traditional dietary pattern with longer LTL—a marker of biologic aging and cardiovascular health [12,13,14]—may shed light on the factors contributing to the observed lower mortality of elderly Costa Ricans relative to other countries [2,4]. The survival advantage of elderly Costa Ricans compared to other countries such as the U.S. is in major part due to reduced cardiovascular mortality. In the 40–89 years age group, heart disease mortality is 35% and 11% lower in Costa Rican men and women, respectively, compared to their U.S. counterparts [2]. Although no individual food group—except grains—was associated with LTL, the traditional Costa Rican dietary pattern is rich in rice and beans. In particular, beans—a major source of protein and fiber in Costa Rican adults [6]—may have cardio-protective effects. The Costa Rica Heart Study (CRHS) found that intake of at least one-serving of beans per day was associated with about 38% lower risk of non-fatal myocardial infarction [6], and high intake of beans was correlated with a beneficial cardio-metabolic profile including lower blood pressure and plasma triglyceride levels, and higher HDL-cholesterol levels [5]. In addition, in a small cross-over intervention study, consumption of pinto bean resulted on reduction of serum total cholesterol and LDL-cholesterol levels [34], and data from the National Health and Examination Survey (NHANES) showed that bean intake was associated with lower body weight and a smaller waist size [35]. Although we did not find that intake of beans was associated with LTL, our results suggest that a traditional Costa Rican dietary pattern—which is rich in rice and beans—may contribute to the extended longevity in elderly Costa Ricans.

Our study has several strengths, such as prospective study design, nationally representative of the population of elderly Costa Ricans, and a relatively big sample size. We were also able to control for a series of socioeconomic and demographic factors. We also note some limitations. Although the abbreviated FFQ (27 food items) was designed to explain most of the variation of macronutrients intake, it may fail to capture most of the variation of food intake in Costa Rican adults. For example, the used FFQ did not ask about refined vs. whole grains. Regarding legumes, the FFQ only asked about red and black beans and other types of beans (e.g., soybeans, peas) were not included in the FFQ. However, we note that the two identified dietary patterns explained about 16% of the total food-intake variation in CRELES. This result is similar to a previous study of myocardial infarction in middle-aged Costa Ricans, which identified two major dietary patterns explaining about 15% of total food-intake variation using a 135 food items FFQ [22]. These results suggest that even though the reduced CRELES FFQ has limitations to measure intake of individual foods, it was able to capture biological relevant variation at the dietary pattern level in the elderly Costa Rican population. Another limitation is that we did not correct our P values for multiple testing even though we evaluated two dietary patterns and several food groups in relation to LTL. We note that these different tests do not represent independent hypotheses as the selected food groups are part of a dietary pattern and, by definition, correlated among them. Therefore, a Bonferroni-like correction would be over-conservative. We decide then to present our results without multiple-testing correction and make interpretations with caution taking into account relevant literature. We also note the cross-sectional nature of our major findings, that limit our ability to draw causal conclusions of the relation between diet and LTL. In addition, our results may be not necessarily generalizable to other populations. Additionally, although our sample size was relatively large, our study may be underpowered to identify weak to moderate associations of diet with LTL. At last, we cannot completely rule out the presence of residual confounding. The traditional Costa Rican dietary pattern was more prevalent in rural parts of the country and may be part of an overall rural lifestyle. It is possible that confounding due to rural lifestyle factors was not adequately controlled for. In summary, results from the present study suggests that food factors may be associated with LTL in elderly Costa Ricans. In particular, a traditional Costa Rican dietary pattern was associated with longer LTL and may in part contribute to the extended longevity of elderly Costa Ricans.

## Figures and Tables

**Table 1:** Factor loadings of food groups with the two major dietary patterns.

Foods or Food Groups	Factor 1	Factor 2
Beverages, alcoholic, liquor	−0.06	0.21
Beverages, alcoholic	0.00	0.18
Beverages, soft drinks	0.02	0.29
Condiment	−0.04	0.34
Dairy products	−0.42	−0.13
Dressings	−0.48	−0.01
Eggs	0.07	−0.03
Fruit	−0.43	−0.06
Fruit juice	0.00	−0.11
Grains	0.64	−0.11
Legumes	0.67	−0.24
Meat, chicken	0.24	0.24
Meat, fish	−0.39	0.11
Meat, red	−0.07	0.33
Oil, soybean	0.15	0.61
Oil, palm	0.13	−0.56
Snacks	−0.02	0.22
Spread	0.01	0.30
Sugar	0.27	−0.01
Sweets and desserts	−0.37	−0.02
Fried food	0.00	0.35
% explained variance (food intake)	9.15	7.18

**Table 2:** Characteristics of the participants: overall and according to quintiles of dietary patterns.

Characteristic	Overall (N = 909)	Factor 1			Factor 2		
		Q1	Q3	Q5	Q1	Q3	Q5
Age, y	76.6	77.9	75.6	75.4 *	77.3	76.9	77.3
Women, %	54.2	64.1	61.5	39.0 ***	48.1	54.4	58.2 *
Telomere length, bp (wave 1)	5423.1	5367.6	5395.5	5443.8 *	5448.6	5424.6	5444.5
Telomere length, bp (wave 2)	5465.3	5473.5	5408.2	5523.9	5471.7	5421.7	5474.4
Nicoya, %	18.4	7.2	23.1	22.0 ***	28.7	18.7	13.7 ***
Rural, %	41.7	21.0	44.5	58.8 ***	56.9	46.7	25.8 ***
Education 11+ y, %	7.4	22.1	3.8	3.3 ***	3.3	4.9	7.7
Current smoking, %	7.3	3.9	7.7	9.9 **	5.5	8.3	4.9
Current drinking, %	28.1	34.3	28.6	25.8	19.3	26.9	33.0 **
Regular exercise, %	25.9	28.2	23.1	26.4	17.2	22.0	32.4 **
Diabetes, %	31.7	36.7	30.4	28.2 *	26.3	33.9	35.9
Self-rated health good or better, %	50.5	60.2	47.8	50.6	43.7	47.3	55.5 *
Household wealth high, %	49.8	76.4	48.0	35.2 ***	37.8	50.3	55.6 **
Nutrient intake ^a^							
Energy (kcal/day)	2120	2263	2113	2086	2159	2134	2250
Protein (g/day)	69	69	70	68	67	69	70 **
Carbohydrate (g/day)	305	289	309	317 ***	320	307	291 ***
Saturated fat (g/day)	27	28	27	25 ***	29	26	28
Polyunsaturated fat (g/day)	18	18	18	18	13	19	19 ***
Monounsaturated fat (g/day)	24	31	22	21 ***	22	23	26 ***
Trans fat (g/day)	2.9	2.7	3.0	2.9 **	2.4	3.1	3.3 ***
Cholesterol (mg/day)	249	259	253	217 ***	231	247	261 **
Fiber (g/day)	23	22	23	25 ***	24	24	21 ***

* P < 0.05, ** P < 0.01, *** P < 0.001 for linear trend differences across quintiles of dietary patterns. ^a^ Energy intake was adjusted for age and sex. All other nutrients were further adjusted for energy intake.

**Table 3:** Association of continuous baseline dietary patterns and telomere length (base-pairs) waves 1 and 2 of interviews.

Dietary Pattern	LTL Wave 1		LTL Wave 2		LTL Wave 2 ^1^		∆LTL ^2^	
	Β ^3^ (95% CI)	P	β (95% CI)	P	β (95% CI)	P	β (95% CI)	P
Factor 1 (traditional)								
Basic model ^4^	32.2 (1.5, 62.9)	0.04	9.2 (−22.4, 40.8)	0.57	−8.0 (−35.1, 19.1)	0.56	−23.1 (−53.7, 7.6)	0.14
MV1 ^5^	40.1 (10.9, 69.3)	0.007	30.0 (2.0, 58.0)	0.04	11.0 (−13.9, 35.9)	0.39	−12.8 (−41.4, 15.8)	0.38
MV2 ^6^	42.0 (9.9, 74.1)	0.01	34.7 (3.8, 65.7)	0.03	14.0 (−13.5, 41.4)	0.32	−9.9 (−41.5, 21.6)	0.54
Factor 2								
Basic model	−11.3 (−40.3, 17.7)	0.45	2.3 (−27.6, 32.2)	0.88	8.3 (−17.3, 33.9)	0.53	13.6 (−15.4, 42.5)	0.36
MV1	−9.2 (−36.6, 18.2)	0.51	2.5 (−24.0, 8.9)	0.86	6.1 (−17.3, 29.4)	0.61	10.6 (−16.4, 37.5)	0.44
MV2	−11.4 (−39.9, 17.1)	0.43	−1.8 (−29.1, 25.5)	0.90	3.3 (−20.8, 27.5)	0.79	10.5 (−17.5, 38.4)	0.46

^1^ Adjusting for wave 1 (baseline) telomere length. ^2^ Change of telomere length = LTL wave 2 − LTL wave 1. ^3^ Beta estimates refer to change of telomere length in base-pairs per one-unit increase of dietary pattern. ^4^ Basic model adjusted for age, sex, and energy intake. ^5^ Multivariate model 1 (MV1) = Basic model + season of the year of blood collection, number of years of DNA storage, whether DNA was extracted from fresh blood cells, and lot of the LTL assay. ^6^ Multivariate model 2 (MV2) = MV1 + rural and Nicoya, education level, smoking, alcohol drinking, regular exercise, self-rated health, household wealth, interviewer.

**Table 4:** Joint association analysis of individual food groups (loadings at least 0.3 in absolute value with Factor 1). Beta estimates and 95% confidence intervals (CI) for telomere length (base-pairs) from waves 1 and 2.

Food Group ^2^	Beta ^1^ (95% CI) Per One-Serving/Day Increase							
	LTL Wave 1		LTL Wave 2		LTL Wave 2 ^3^		∆LTL ^4^	
	β (95% CI)	P	β (95% CI)	P	β (95% CI)	P	β (95% CI)	P
Dairy products	−6.3 (−40.3, 27.6)	0.71	−25.5 (−58.3, 7.2)	0.13	−21.8 (−50.7, 7.1)	0.14	−16.7 (−49.9, 16.6)	0.33
Dressings	−106.5 (−233.6, 20.5)	0.10	−20.9 (−143.3, 101.6)	0.74	26.7 (−81.3, 134.8)	0.63	93.4 (−30.9, 217.8)	0.14
Fruit	−6.0 (−44.5, 32.6)	0.76	−9.9 (−47.1, 27.4)	0.60	−9.3 (−42.0, 23.5)	0.58	−5.8 (−43.6, 32.0)	0.76
Grains	43.6 (13.9, 73.3)	0.004	12.8 (−15.8, 41.4)	0.38	−7.6 (−32.9, 17.7)	0.56	−25.4 (−54.5, 3.7)	0.10
Legumes	−5.9 (−42.8, 31.1)	0.76	12.2 (−23.3, 47.7)	0.50	13.4 (−17.9, 44.6)	0.40	9.7 (−26.5, 45.9)	0.60
Meat, fish	67.9 (−173.0, 308.9)	0.58	106.2 (−125.5, 337.9)	0.37	81.1 (−123.1, 285.3)	0.44	7.8 (−227.9, 243.6)	0.95
Sweets and desserts	36.4 (−13.6, 86.4)	0.15	−3.1 (−51.4, 45.2)	0.90	−18.4 (−61.0, 24.2)	0.40	−29.2 (−78.3, 19.9)	0.24

^1^ Adjusted for age, sex, rural and Nicoya residence, energy intake, education level, smoking, alcohol drinking, regular exercise, self-rated health, household, interviewer, season of the year of blood collection, number of years of DNA storage, whether DNA was extracted from fresh blood cells, and lot of the LTL assay. ^2^ Food groups were jointly analyzed in a single multivariate model. ^3^ Adjusted for wave 1 (baseline) telomere length. ^4^ Change of telomere length = LTL wave 2 − LTL wave 1.

## Data Availability

The data presented in this study are openly available in The National Archive of Computerized Data on Aging of the University of Michigan: http://www.icpsr.umich.edu/icpsrweb/NACDA/series/386. Leukocyte telomere data are openly available at https://journals.plos.org/plosone/article?id=10.1371/journal.pone.0223766.

## Supplementary Materials

The following are available online at https://www.mdpi.com/article/10.3390/nu13082585/s1, Table S1: Explained variance of intake of selected nutrients with the CRELES abbreviated food frequency questionnaire (27 food items). Table S2: Food groups in the Costa Rican diet. Figure S1: Telomere length (bp) wave 2 vs. telomere length wave 1 (bp).
