# Supplementary material for: Diet and Leukocyte Telomere Length in a Population with Extended Longevity: The Costa Rican Longevity and Healthy Aging Study (CRELES)
Source: Nutrients. 2021 Jul 28;13(8):2585. doi: 10.3390/nu13082585 (PMC8401744; doi:10.3390/nu13082585)
Supplement: Supplementary file 1 [file nutrients-13-02585-s001.zip › Supplementary Table S1.pdf]

Supplementary Table S1. Explained variance of intake of selected nutrients with the CRELES abbreviated food frequency questionnaire (27 food items)

Nutrient	Explained variance, R^2
Total energy, kcal/d	0.81
Protein, g/d	0.80
Carbohydrates, g/d	0.76
Glycemic load, g/d	0.78
Total fat, g/d	0.85
Saturated fat, g/d	0.84
Monounsaturated fat, g/d	0.88
Polyunsaturated fat, g/d	0.82
Omega-6 fatty acid, g/d	0.81
Omega-3 fatty acid, g/d	0.85
Trans fat, g/d	0.85
Cholesterol, mg/d	0.94
Fiber, g/d	0.76
Alpha-tocopherol, mg/d	0.78
Gamma-tocopherol, mg/d	0.86
Calcium, mg/d	0.84
Alcohol, g/d	0.99

Data from CRELES website https://www.icpsr.umich.edu/icpsrweb/NACDA/series/386
