# Supplementary material for: Diet and Leukocyte Telomere Length in a Population with Extended Longevity: The Costa Rican Longevity and Healthy Aging Study (CRELES)
Source: Nutrients. 2021 Jul 28;13(8):2585. doi: 10.3390/nu13082585 (PMC8401744; doi:10.3390/nu13082585)
Supplement: Supplementary file 1 [file nutrients-13-02585-s001.zip › Supplementary Table S2.pdf]

Supplementary Table S2. Food groups in the Costa Rican diet

Foods or food groups	Food items
Beverages, alcoholic, liquor	Rum, whisky, gin, vodka, ‘guaro’
Beverages, alcoholic	Beer
Beverages, soft drinks	Coke, Pepsi
Condiment	Mayonnaise
Dairy products	Milk, sour cream, fresh white cheese, white and yellow processed cheese
Dressings	Olive oil (as salad dressing, added to bread)
Eggs	Hen eggs
Fruit	Bananas, avocado, mango, oranges
Fruit juice	Fresh fruit drinks (lemonade, berry, pineapple)
Grains	White rice, white bread
Legumes	Black or red beans
Meat, chicken	Chicken with skin
Meat, fish	Fish (scallops, sea bass, other)
Meat, red	Beef, meatballs
Oil, soybean	Soybean oil used for cooking
Oil, palm	Hardened palm oil (manteca) used for cooking
Snacks	Potato chips or corn chips
Spread	Margarine with bread or with meals
Sugar	Sugar added to beverages and meals
Sweets and desserts	Cookies, baked goods, pastries, sweet rolls, coffee cake
Fried food	Fried foods prepared away from your home
