# Supplementary figures and images for: Diet and Leukocyte Telomere Length in a Population with Extended Longevity: The Costa Rican Longevity and Healthy Aging Study (CRELES)
Source: Nutrients. 2021 Jul 28;13(8):2585. doi: 10.3390/nu13082585 (PMC8401744; doi:10.3390/nu13082585)

**Supplementary Figure 1**

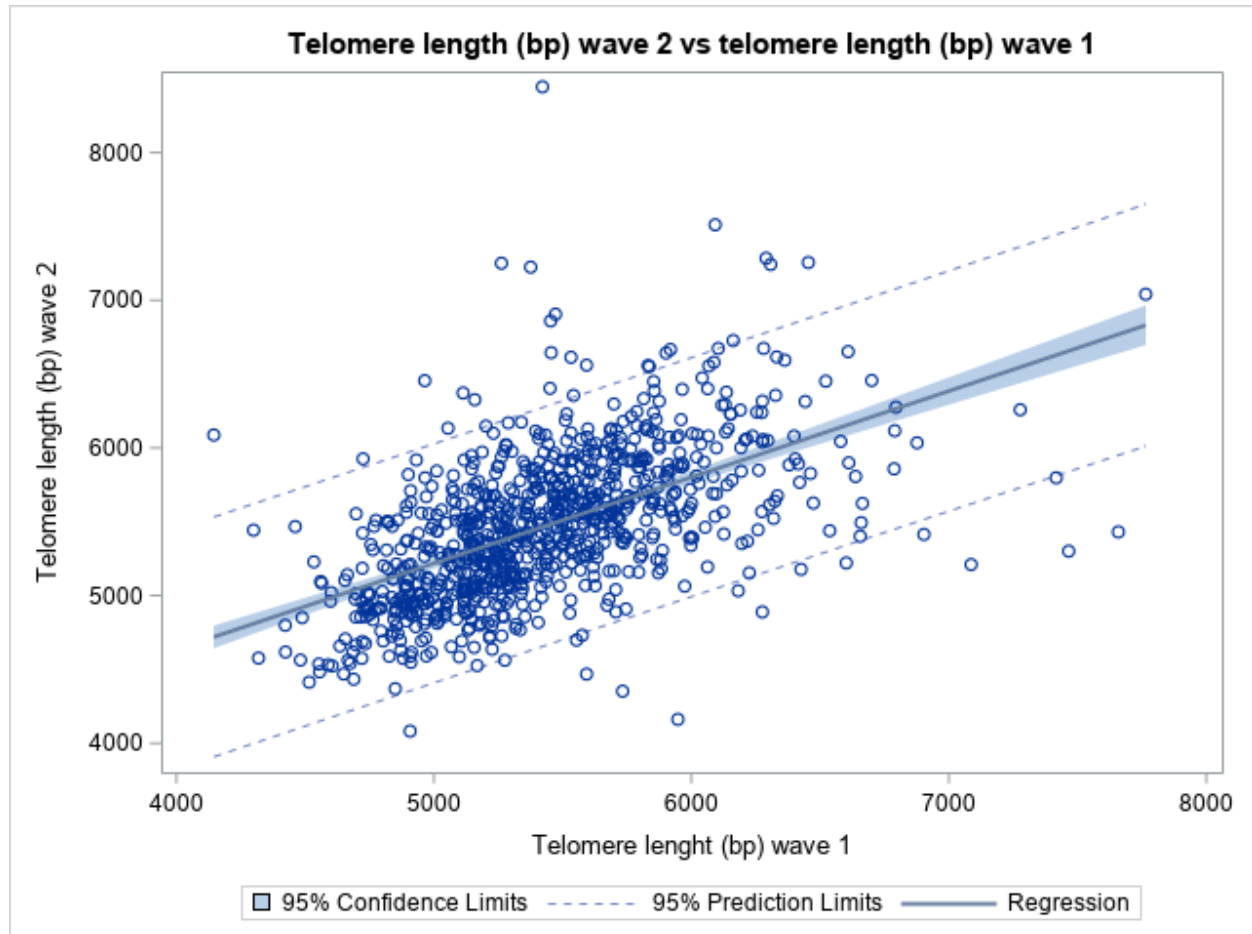

Supplement: Supplementary file 1 [file nutrients-13-02585-s001.zip › Supplementary Figure 1.pdf]
